# Targeting Oligosaccharides and Glycoconjugates Using Superselective Binding Scaffolds

**DOI:** 10.1002/adfm.202002298

**Published:** 2020-05-28

**Authors:** Stefano Tommasone, Yazmin K. Tagger, Paula M. Mendes

**Affiliations:** ^1^ School of Chemical Engineering University of Birmingham Edgbaston Birmingham B15 2TT UK

**Keywords:** binding scaffolds, glycan recognition, glycoproteins, oligosaccharide recognition, superselective binding, synthetic materials

## Abstract

Recognition of oligosaccharides is associated with very limited specificity due to their strong solvation in water and the high degree of subtle structural variations between them. Here, oligosaccharide recognition sites are created on material surfaces with unmatched, binary on–off binding behavior, sharply discriminating a target oligosaccharide over closely related carbohydrate structures. The basis for the superselective binding behavior relies on the highly efficient generation of a pure, high order complex of the oligosaccharide target with synthetic carbohydrate receptor sites, in which the spatial arrangement of the multiple receptors in the complex is preserved upon material surface incorporation. The synthetic binding scaffolds can easily be tailored to recognize different oligosaccharides and glycoconjugates, opening up a realm of possibilities for their use in a wide field of applications, ranging from life sciences to diagnostics.

## Graphical abstract

A unique modular, synthetic strategy allows for the creation of artificial binding sites with precise spatial positioning of multiple carbohydrate receptors, enabling the remarkable ability to distinguish a target oligosaccharide over closely related carbohydrate structures. The synthetic binding scaffolds can easily be tailored to bind not only specifically oligosaccharides in free form but also when they are linked to proteins as glycoconjugates.

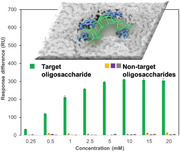

## 1 Introduction

**Figure 1:**
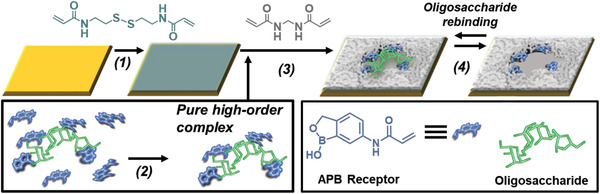
Method for creating synthetic materials with superselective oligosaccharide recognition. 1) Acrylamide‐terminated monolayer formation using *N,N′*‐bis(acryloyl)cystamine; 2) pure, high‐order oligosaccharide: 5‐acrylamido‐2‐(hydroxymethyl)phenylboronic acid cyclic monoester (APB) complex formation; 3) fixation of the complex on the surface and construction of molecular scaffold around the oligosaccharide template using *N,N*′‐methylenebisacrylamide; 4) removal of the oligosaccharide template.

Oligosaccharides, which often occur as glycoconjugates, play essential roles within a multitude of biological processes, including fertilization, cell differentiation, cell signaling, and host–pathogen interactions.^[^ ^1^, ^2^, ^3^, ^4^ ^]^ Furthermore, they are emerging as important biomarkers for a wide range of diseases, including immune deficiencies, hereditary disorders, neurodegenerative and cardiovascular diseases, and many types of cancers.^[^ ^5^, ^6^, ^7^ ^]^ Thus, materials with highly specific oligosaccharide recognition are key for advancing glycobiology research and producing new opportunities to diagnose and treat diseases. However, the approaches used today, that rely on anticarbohydrate antibodies,^[^ ^8^ ^]^ lectins,^[^ ^9^ ^]^ aptamers,^[^ ^10^ ^]^ and synthetic carbohydrate receptors,^[^ ^11^ ^]^ are limited in their capabilities to discriminate between a large repertoire of carbohydrate structures, including closely related isomers.^[^ ^12^ ^]^ For instance, natural and recombinant lectins exhibit specificity only toward a particular carbohydrate motif or structural feature and are available in a very limited number when compared with the striking variety of oligosaccharide structures.^[^ ^13^ ^]^ On the other hand, oligosaccharides are poorly immunogenic, posing major hurdles in the development of highly selective anticarbohydrate antibodies.^[^ ^14^ ^]^ Examples of aptamers that evolved to recognize oligosaccharides are scarce owing to the limited number of noncovalent interactions that can be harnessed between carbohydrates and oligonucleotides.^[^ ^15^, ^16^ ^]^ Synthetic carbohydrate receptors, including boronic acid moieties, which form reversible covalent complexes with diols, have been combined with molecular imprinting to obtain carbohydrate binding sites on polymer matrices.^[^ ^17^, ^18^ ^]^ However, the available synthetic approaches are incapable to encode the binding sites with precise molecular complementarity to target oligosaccharides. Here, we report on a modular synthetic approach that harnesses both the construction of high‐yield, complex oligosaccharide–synthetic carbohydrate receptor assemblies and the precise generation of surface‐confined templated binding sites (**Figure** **1**), thereby creating recognition sites of unparalleled oligosaccharide discrimination. Benzoboroxoles are employed as carbohydrate receptors since, in contrast to their boronic acids analogs, benzoboroxoles can bind nonreducing hexopyranosides at pH values compatible with biological systems.^[^ ^19^ ^]^

## 2 Results and Discussion

Initially, we demonstrated the feasibility of creating stable, high‐order complexes between oligosaccharides and benzoboroxoles using three model oligosaccharides, namely, stachyose **1**, nystose **2**, and verbascose **3**. Optimum complexation conditions were achieved by stirring for 24 h a mixture of an excess of 2‐(hydroxymethyl)phenylboronic acid cyclic monoester **4** (8.0 equivalents per sugar unit) and oligosaccharide in dioxane:acetonitrile (6:1 v/v) at 90 °C. These conditions provided the optimal compromise for both solubility and reaction temperature. An indirect method has been devised, using partial chemical benzoylation and mass spectrometry analysis, for obtaining a relative estimate of the different high‐order complexes formed (Figure S1, Supporting Information). Following complexation, the resulting complex was treated with benzoyl chloride in pyridine for 5 h, in order to functionalize the OH groups not involved in any bond with the boron. Afterward, the boronate esters were hydrolyzed by treatment with 1 m aqueous solution of sorbitol/Na_2_CO_3_ and EtOAc, and the product was finally recovered by several washings with EtOAc.^[^ ^20^ ^]^ Together with the known binding mechanism of boronic acid derivatives with diols,^[^ ^19^, ^21^ ^]^ the analysis of the resulting product gave us insight into the efficiency of the complexation and the structure of the complexes formed (more details in Figures S2–S8 in the Supporting Information). These analyses demonstrated that the highest‐order complexes were preferentially formed (**Table** **1**). For instance, while stachyose **1** can interact with benzoboroxoles to form complexes with stoichiometry ranging from 1:1 up to 1:4, the results indicate that stachyose was able to form complexes with benzoboroxoles in high 1:3 and 1:4 stoichiometric ratios, with a greater proportion of 1:4 (80%) than 1:3 (20%) complex. Benzoboroxoles usually bind fructose units in positions 2 and 3. However, according to the structure of nystose **2**, the hydroxyls in positions 2 are not accessible since they are involved in the formation of glycosidic bonds. Nevertheless, with nystose, we observed the formation of high‐order complexes, as we found evidence of a 1:3 adduct and some 1:4 (maximum degree of complexation). A possible explanation is that the binding takes place via the OH in position 3 and 6, which are in a sin‐periplanar relationship.^[^ ^22^ ^]^ This demonstrates that our approach can push the formation of boronate esters even when the conditions are less favorable, as well as being of general applicability since it affords high‐order complexes with oligosaccharides with different sizes and stereochemistry.

**Table 1:** Degree of complexation of different oligosaccharides, stachyose 1, nystose 2, and verbascose 3 with 2‐(hydroxymethyl)phenylboronic acid cyclic monoester 4. Relative ratios (%) of complexes derived by the MALDI spectra of the products isolated after column chromatography following the approach in Figure S1 (Supporting Information)

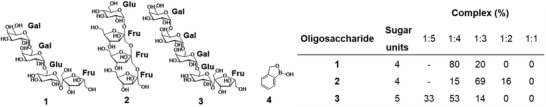

The construction of the oligosaccharide binding scaffolds was initiated by immersing clean gold substrates in a 0.1 × 10^−3^ m ethanolic solution of *N,N′*‐bis(acryloyl)cystamine with 2% trifluoracetic acid (TFA) for 24 h. The formation of the acrylamide‐terminated self‐assembled monolayers (SAMs) was confirmed by contact angle (advancing and receding contact angles of 78 ± 1° and 62 ± 2°, respectively), ellipsometry (thickness of 0.56 ± 0.06 nm), and X‐ray photoelectron spectroscopy (XPS). XPS peaks (Figure S9, Supporting Information) account for the presence of C (1s), O (1s), N (1s), and S (2p), with the binding energies of the S (2p) peaks at 161.6 and 162.8 eV, indicating the chemisorption of the *N,N′*‐bis(acryloyl)cystamine on the gold surface through S—Au bonds.

**Figure 2:**
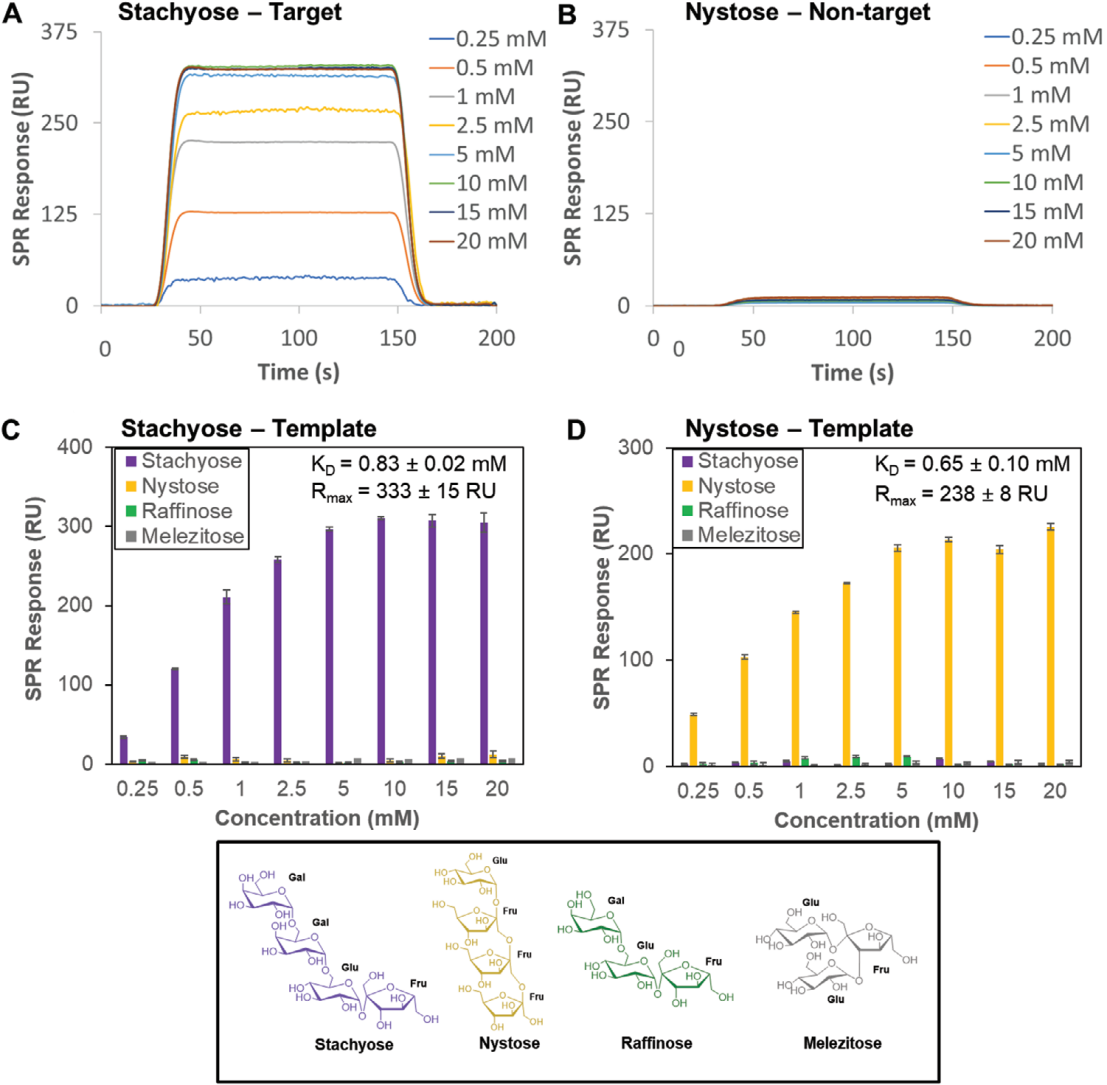
SPR sensorgram traces performed on binding scaffolds prepared on SPR chips using stachyose as a template and different concentrations of A) stachyose and B) nystose flowed over the surface at pH 7.4. SPR responses at equilibrium against the concentration of injected oligosaccharides, stachyose, nystose, raffinose and melezitose (shown at the bottom) using C) stachyose‐binding scaffolds and D) nystose‐binding scaffolds, from which *K*_D_ and *R*_max_ values have been obtained.

We initially created oligosaccharide‐binding scaffolds using stachyose as the template. Following its complexation with 5‐acrylamido‐2‐(hydroxymethyl)phenylboronic acid cyclic monoester (APB; Figure 1), the complex and free APB were separated by precipitation followed by centrifugation. This protocol enabled us to remove the excess of unreacted APB, which otherwise would have had a detrimental effect in creating precise recognition sites for oligosaccharides. The binding scaffolds were prepared by grafting simultaneously *N,N′*‐methylenebisacrylamide (MBA) and the high‐order complexes between stachyose and APB onto the acrylamide‐terminated SAMs for 15 min. Following dissociation of the oligosaccharide from the surface under acidic conditions, the binding affinity and selectivity of the stachyose‐binding scaffolds was evaluated using surface plasmon resonance (SPR) spectroscopy. Stachyose and three structurally related oligosaccharides, namely, nystose, raffinose, and melezitose, were employed to evaluate the selectivity of the binding scaffolds. The SPR sensorgrams of the binding scaffolds prepared using stachyose as a template reveal a striking difference in binding between the target stachyose oligosaccharide (**Figure** **2**A) and nontarget oligosaccharides (e.g., nystose; Figure 2B), with the formed binding sites allowing for a great degree of subtlety in recognizing stachyose. SPR binding analysis show that stachyose binds the stachyose‐binding scaffolds with a dissociation constant (*K*_D_) of 0.83 × 10^−3^ m, while no or negligible binding was observed for the nontarget oligosaccharides (Figure 2C). In creating a nystose‐binding scaffold, the selectivity is reversed and instead the recognition sites can only bind nystose, with a *K*_D_ of 0.65 × 10^−3^ m (Figure 2D).

The dissociation constants of benzoboroxoles for monosaccharides are largely dependent on their structure, with reported *K*_D_ values for fructose, glucose and methyl α‐d‐galactopyranoside of 2.95, 32.3, and 34.5 × 10^−3^ m, respectively.^[^ ^23^ ^]^ Thus, the surface‐confined binding scaffolds resulted in 5–50‐fold higher binding affinity for the target oligosaccharide as compared to monosaccharides. This behavior suggests that multivalent interactions are occurring between multiple benzoboroxoles receptors incorporated in the binding site with multiple hydroxyl groups within the oligosaccharide chain. While the binding affinity is comparable to that of oligosaccharide antibodies and lectins with dissociation constants in the low mm range,^[^ ^12^, ^24^ ^]^ our oligosaccharide recognition sites exhibit an unprecedented binary on–off oligosaccharide binding behavior.

**Figure 3:**
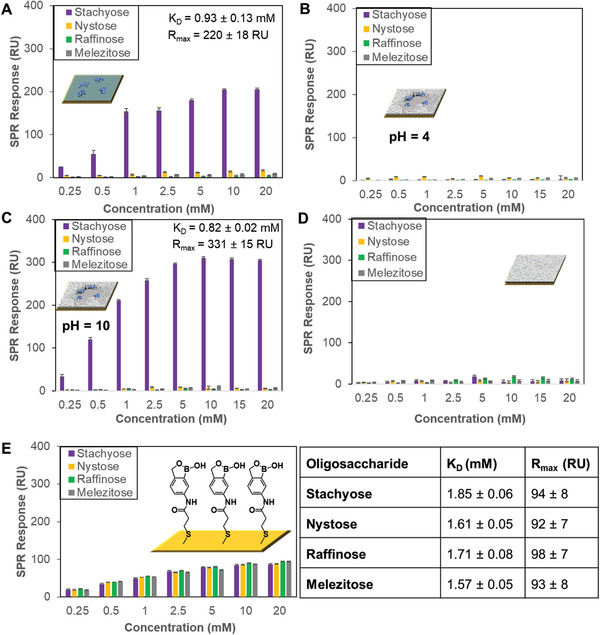
SPR responses at equilibrium against the concentration of injected oligosaccharides, stachyose, nystose, raffinose, and melezitose. A) Acrylamide‐terminated SAMs with only high‐order complexes between stachyose and APB grafted on it (i.e., absence of MBA) using pH 7.4 oligosaccharide solutions. B) pH 4 and C) pH 10 oligosaccharide solutions were employed to run SPR on stachyose‐binding scaffolds. D) Surfaces prepared by copolymerizing MBA onto the acrylamide‐terminated SAMs using pH 7.4 oligosaccharide solutions. E) Benzoboroxole‐terminated SAMs using pH 7.4 oligosaccharide solutions. The table illustrates the *K*_D_ and *R*_max_ values obtained for the different oligosaccharides used.

The achieved maximum binding capacity (*R*_max_) for the stachyose‐ and nystose‐binding scaffolds was in the range of 0.2–0.3 ng mm^−2^ (100 response units (RUs) = 0.1 ng mm^2[^25^]^), corresponding to 1 oligosaccharide per 6–4 nm^2^. Assuming a footprint of approximately 2–3 nm^2^ for a tetrasaccharide,^[^ ^26^ ^]^ an estimated 50% surface coverage by oligosaccharide can be achieved. The remaining surface area comprises crosslinked MBA, which defines the pocket shape and size of the oligosaccharide used as template, enhancing to some extent its binding affinity. A control surface obtained by grafting only high‐order complexes between stachyose and APB onto the acrylamide‐terminated SAMs for 15 min (i.e., absence of MBA) has led to similar superselectivity for stachyose (**Figure** **3**A), but a slightly higher *K*_D_ value of (0.93 ± 0.13) × 10^−3^ m. The MBA is a crosslinking agent that allows building a molecular scaffold around the template. Apart from creating a shape complementary to the template, MBA can provide additional weak interactions with carbohydrates (i.e., hydrogen bonds) which could enhance the affinity for the target template. However, in our case only a slightly increase in the binding affinity was observed (*K*_D_ of 0.83 × 10^−3^ m with MBA, Figure 2C vs 0.93 × 10^−3^ m without MBA, Figure 3A). This is probably because either the thickness of the polymer layer is too small and does not allow, the MBA to have a significant effect on the overall binding or the crosslinker does not make the surface rigid enough to affect the properties of the binding site.

The presence of the benzoboroxole receptors in the recognition site is crucial to establish the selective binding for the target oligosaccharide. At pH 7.4, the binding scaffolds showed higher binding for the target oligosaccharide such as stachyose due to the benzoboroxole moieties forming boronate esters with stachyose, whereas no binding occurred at pH 4 (Figure 3B) since boronate ester formation is less favorable in acidic conditions. While acidic conditions disrupt binding, the stachyose‐binding scaffolds displayed similar binding behavior towards stachyose at pH 7.4 (*K*_D_ = 0.83± 0.02, Figure 2C) and pH 10 (*K*_D_ = 0.82± 0.02, Figure 3C). Furthermore, control experiments involving only the copolymerization of MBA onto the acrylamide‐terminated SAMs have led to negligible binding to all the oligosaccharides (Figure 3D), indicating that selectivity arises from the binding pockets containing the suitably spatially arranged benzoboroxole receptors.

Our findings provide evidence of the importance of precise and multivalent spatial pattern recognition to achieve superselective oligosaccharide binding. When we take into consideration that stachyose is a higher homolog of raffinose and an all‐or‐nothing binding occurs between them, it indicates that the nature of the superselective behavior is likely associated with a threshold in binding stability. The precise spatial arrangement of the receptors promotes the establishment of multiple interactions with the target oligosaccharide, stabilizing the binding event with consequent enhanced *K*_D_ values, which is otherwise not possible with oligosaccharides that do not match the binding site. Although raffinose could potentially fit the binding sites of stachyose‐binding scaffolds, it is probably not able to establish enough interactions to overcome the energetic requirements to reach an observable binding. This interpretation is supported by control experiments, wherein a surface comprising a monolayer of benzoboroxoles show similar binding for stachyose, nystose, raffinose and melezitose (Figure 3E), with *K*_D_ values in the range (1.58–1.86) × 10^−3^ m. The presence of a high density of benzoboroxoles on the surface is not able to provide a specific spatial arrangement to regulate binding stability of target and nontarget oligosaccharides, with these surfaces allowing the binding of all the oligosaccharides. The superselectivity of our system could find some analogy in a mechanism previously proposed,^[^ ^27^ ^]^ where the binding energy is not a linear function of the number of bonds but grows more rapidly. In fact, we were able to distinguish between a ligand that can form three bonds (raffinose) and one that can form four (stachyose), with an all‐or‐nothing behavior. However, we believe that the superselectivity of our system also accounts for an additional contribution, which could be related to geometrical factors. A fine control of the shape complementarity of the binding site could explain why we can also discriminate between oligosaccharides with the same number of ligands (stachyose and nystose). Another point to consider is that benzoboroxoles have different binding affinities for different carbohydrates (Gal, Glu, and Fru), therefore each sugar unit of the oligosaccharides interacts with the receptors in a different way.

**Figure 4:**
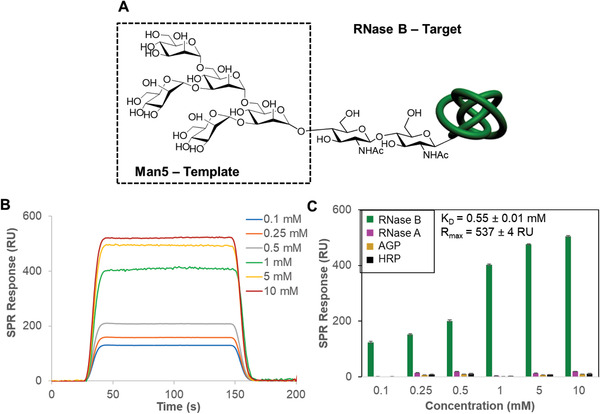
A) The oligosaccharide structure of the Man5 glycoform of RNase B, of which Man5 was used as a template for the generation of the binding scaffolds. The Man6–Man9 RNase B glycoforms contain further mannose units, which are added to the outer three mannose residues in Man5. B) SPR sensorgram traces performed with Man5‐binding scaffolds on the SPR chip and different concentrations of RNase B flowed over the surface. C) SPR responses at equilibrium against the concentration of injected protein, RNase B, RNase A, AGP, and HRP using Man5‐binding scaffolds, from which *K*_D_ and *R*_max_ values have been obtained.

The power of the methodology was further demonstrated by the capability of the novel binding scaffolds to bind specifically their glycoconjugates (**Figure** **4**). Ribonuclease B (RNase B) contains a single glycosylation site of high‐mannose type with 5–9 mannose residues, Man5–Man9.^[^ ^28^ ^]^ Binding scaffolds using Man5 as the template were shown to bind only RNase B and not the nonglycosylated RNase form, RNase A, and two highly glycosylated glycoproteins, α1‐acid glycoprotein (AGP) and horseradish peroxidase (HRP). AGP (45% glycosylation) possesses complex‐type glycans that are strongly sialylated,^[^ ^29^ ^]^ while HRP (21% glycosylation) consists predominantly of the oligosaccharide (Xyl)Man3(Fuc)GlcNAc2, containing only low levels of the high mannose‐type glycan.^[^ ^30^ ^]^

## 3 Conclusion

In conclusion, a unique modular strategy, which harnesses supramolecular assembly and well‐controlled chemistry, was developed to create robust and highly reproducible template‐induced oligosaccharide recognition sites on synthetic scaffolds. Our findings show that our approach has a remarkable ability to deliver synthetic receptors capable of highly specifically targeting oligosaccharides whether they occur in free form or as components of glycoproteins. These results go beyond the scope of the oligosaccharides described here, with the modularity of the synthetic strategy lending itself to adaptivity and incorporation into technologies for diagnostics, biotechnology, and glycobiology research.

## Conflict of Interest

A patent has been filled by the University of Birmingham related with the modular, synthetic strategy reported in this article under WO2015/118294. P.M.M is an author on the patent. The other authors declare that they have no competing interests.

## Supporting information

Supporting Information
