# Supplementary material for: Targeting Oligosaccharides and Glycoconjugates Using Superselective Binding Scaffolds
Source: Adv Funct Mater. 2020 May 28;30(31):2002298. doi: 10.1002/adfm.202002298 (PMC7405978; doi:10.1002/adfm.202002298)
Supplement: Supplementary file 1 — Supporting Information [file ADFM-30-2002298-s001.pdf]

Copyright WILEY-VCH Verlag GmbH & Co. KGaA, 69469 Weinheim, Germany, 2020.

ADVANCED FUNCTIONAL MATERIALS

Supporting Information

for *Adv. Funct. Mater.,* DOI: 10.1002/adfm.202002298

Targeting Oligosaccharides and Glycoconjugates Using Superselective Binding Scaffolds

*Stefano Tommasone, Yazmin K. Tagger, and Paula M. Mendes**

Supporting Information

Targeting oligosaccharides and glycoconjugates using superselective binding scaffolds

Stefano Tommasone,[1,]† Yazmin K. Tagger,[1,]† Paula M. Mendes[1,]*

[1]School of Chemical Engineering, University of Birmingham, Edgbaston, Birmingham, B15 2TT, UK.

*Correspondence to: p.m.mendes@bham.ac.uk

† These authors contributed equally to this work.

Table of contents

1. Materials S2
2. Synthetic procedures S2
3. Complex formation and indirect approach to access degree of complexation S4
4. Mass spectrometry analysis S5
5. Self-assembled monolayer formation and characterization S11
6. Formation of binding scaffold and surface plasmon resonance studies S12

1. Materials

Commercially available solvents were purchased from Sigma-Aldrich and used without further purification. All the chemicals were purchased from Sigma-Aldrich with the exception of Verbascose, purchased from Carbosynth Limited and 5-amino-2-(hydroxymethyl)phenylboronic acid cyclic monoester, purchased from Tokyo Chemical Industry UK Ltd. RNase B from bovine pancreas, RNase A from bovine pancreas, α1-acid glycoprotein from bovine plasma and HRP from horse radish root were all purchased from Sigma Aldrich. ^{1}H and ^{13}C NMR spectra were recorded on a Bruker AVIII400 NMR spectrometer at 400 MHz and 101 MHz respectively, at room temperature. Thin layer chromatography (TLC) was performed using commercially available Macherey-Nagel aluminium backed plates coated with a 0.20 mm layer of silica gel 60 Å with fluorescent indicator UV_{254}. TLC plates were visualised using either ultraviolet light of 254 nm wavelength or by staining with *p*-anisaldehyde solution. Silica gel column chromatography was carried out using Sigma-Aldrich 60 A silica gel (35-70 µm). Mass spectra were recorded with Waters Xevo G2-XS using electrospray ionization mode and with Synapt G2-Si (Waters Ltd) using Nanospray or MALDI Micro Mx (Waters Micro mass Ltd) ionization modes. Polycrystalline gold substrates were purchased from George Albert PVD, Germany. The gold substrates consist of a 100 nm gold layer deposited onto glass, which are covered with a 5 nm layer of chromium as an adhesion layer. These gold substrates were used for ellipsometry, XPS and contact angle analysis. The polycrystalline gold substrates which were employed in SPR experiments were purchased from Reichert Technologies, USA. The gold substrates consist of 49 nm gold with 1 nm chromium.

2. Synthetic procedures

2.1 Synthesis of 5-acrylamido-2-(hydroxymethyl)phenylboronic acid cyclic monoester (APB)[1]

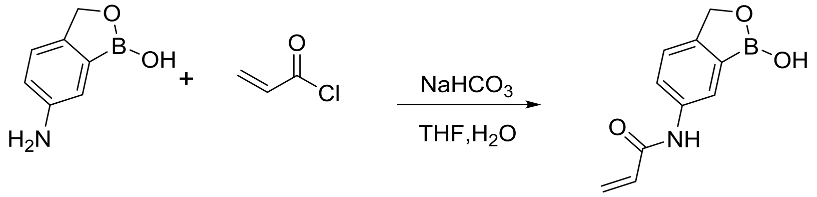

5-Amino-2-(hydroxymethyl)phenylboronic acid cyclic monoester (0.50 g, 3.35 mmol) was dissolved in a mixture of THF:H_2O (1:1, 11.6 ml). The solution was cooled with an ice-bath and sodium carbonate (1.12 g, 13.40 mmol) and acryloyl chloride (0.54 ml, 6.71 mmol) were then added. The reaction mixture was stirred at room temperature for 5 h. The solvent was removed under reduced pressure and the product was recovered by crystallization from water:methanol (0.30 g, y = 44%). The NMR analysis was in agreement with the literature.[1] ^{1}H NMR (400 MHz, 298K, DMSO-d6): δ 4.93 (s, 2H), 5.75 (dd, J_1 = 10.0 Hz, J_2 = 2.0 Hz, 1H), 6.26 (dd, J_1 = 16.8 Hz, J_2 = 2.0 Hz, 1H), 6.46 (dd, J_1 = 16.8 Hz, J_2 = 10.0 Hz, 1H), 7.35 (bd, J_1 = 8.0 Hz, J_2 = 0.4 Hz, 1H) 7.69 (dd, J_1 = 8.4 Hz, J_2 = 2 Hz, 1H), 8.06 (s,1H), 9.22 (s,1H), 10.19 (s,1H).

2.2 Synthesis of control SAM molecule (5) - 3,3'-disulfanediylbis(*N*-phenylpropanamide)[2]

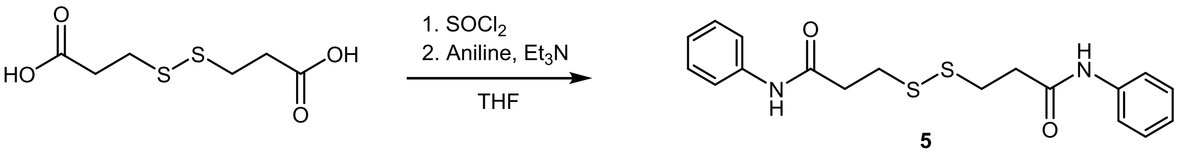

5

A solution of 3,3'-dithiopropionic acid (500 mg, 2.38 mmol) in THF (5 ml) was cooled in an ice bath and $SOCl_2$ (0.67 ml, 9.27 mmol) was added dropwise. The reaction mixture was stirred overnight at room temperature. After cooling the solution with an ice bath, aniline (0.84 ml, 9.27 mmol) and Et_3N (1.29 ml, 9.27 mmol) were slowly added. The reaction mixture was allowed to warm at room temperature and stirred for 4 h. Water was added to quench the reaction and the product was extracted with CH_2Cl_2. The organic phase was washed with water, dried over $MgSO_4$ and concentrated. The product was purified by column chromatography with silica gel (CH_2Cl_2:MeOH, 98:2) (73 mg, yield = 8%). ^{1}H NMR (400 MHz, 298K, DMSO-d_6) δ: 2.74 (t, J = 6.8 Hz, 4H), 3.01 (t, J = 7.2 Hz, 4H), 7.03 (t, J = 7.2 Hz, 2H), 7.30 (t, J = 7.6 Hz, 4H), 7.58 (d, J = 7.6 Hz, 4H), 10.02 (s, 2H); ^{13}C NMR (101 MHz, 298K, DMSO-d_6) δ: 33.5, 36.0, 119.1, 123.1, 128.7, 139.1, 169.1.

2.3 Synthesis of benzoboroxole terminated SAM molecule (6) - 3,3'-disulfanediylbis(N-(1-hydroxy-1,3-dihydrobenzo[c][1,2]oxaborol-6-yl)propanamide)

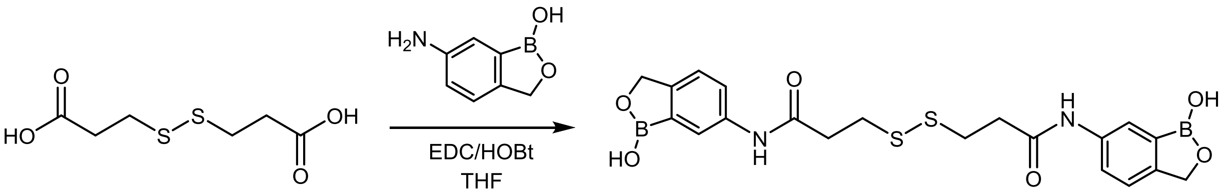

6

3,3'-dithiopropionic acid (58 mg, 0.28 mmol) was dissolved in THF (2.8 ml). EDC (128 mg, 0.67 mmol) and HOBt (90 mg, 0.67 mmol) were added and the reaction mixture was stirred at room temperature for 30 min. Afterwards, 5-amino-2-(hydroxymethyl)phenylboronic acid cyclic monoester (100 mg, 0.67 mmol) was added and the reaction mixture was stirred overnight. The solvent was then removed under reduced pressure and water (10 ml) was added, affording a precipitate that was filtered and dried. The crude was suspended in CH_2Cl_2 and the precipitated was filtered and dried. Finally, the product was suspended in methanol and the white solid obtained was filtered and dried, affording the pure product (55 mg, yield = 42%). ^{1}H NMR (400 MHz, 298K, DMSO-d_6) δ: 2.76 (t, J = 6.4 Hz, 4H), 3.03 (t, J = 6.4 Hz, 4H), 4.92 (s, 4H), 7.31 (d, J = 8 Hz, 2H), 7.61 (d, J = 7.6 Hz, 2H), 7.99 (s, 2H), 9.22 (s, 2H), 10.06 (s, 2H); ^{13}C NMR (101 MHz, 298K, DMSO-d_6) δ: 33.6, 36.0, 69.7, 121.0, 121.5, 122.3, 137.9, 148.6, 169.0. ESI(+) MS *m/z* 495.10 $[M-Na]^+$. HRMS (ESI) *m/z* $[M-Na]^+$ calcd for $C_{20}H_{22}B_2N_2O_6S_2Na$ 495.1010, found 495.1009.

3. Complex formation and indirect approach to access degree of complexation

The oligosaccharide (20.0 mg, 0.030 mmol for stachyose and nystose, 0.024 mmol for verbascose) and 2-(hydroxymethyl)phenylboronic acid cyclic monoester (8.0 eq/sugar unit, 128 mg, 0.96 mmol) were suspended in a mixture of dioxane (2.4 ml) and acetonitrile (0.4 ml). For practical purposes, the commercially available 2-(hydroxymethyl)phenylboronic acid cyclic monoester rather than the synthetic derivative 5-acryalmido-2-(hydroxymethyl)phenylboronic acid cyclic monoester was used. This allowed us to scale up the process and work with a more tangible amount of sugar. The reaction mixture was stirred at 90 ºC for 24 h under argon atmosphere in presence of activated molecular sieves 3Å. The solvent was removed under reduced pressure and the crude was dissolved in pyridine (0.5 ml). Benzoyl chloride (50 µl, 0.41 mmol) was added at 0 ºC and the reaction mixture was stirred at room temperature for 5 h. The reaction mixture was diluted in toluene and filtered through celite. The solvent was removed under reduced pressure and the crude was dissolved in a mixture of EtOAc (5 ml) and a solution of 1 M sorbitol/Na_2CO_3 (5 ml) and stirred for 1 h. The two phases were then separated and the aqueous layer was washed three times with EtOAc. The organic phases were combined, dried over $MgSO_4$ and concentrated under reduced pressure. The crude was then purified by column chromatography on silica gel (DCM:MeOH 99:1 – 90:10). The fractions collected were analysed by MALDI-MS.

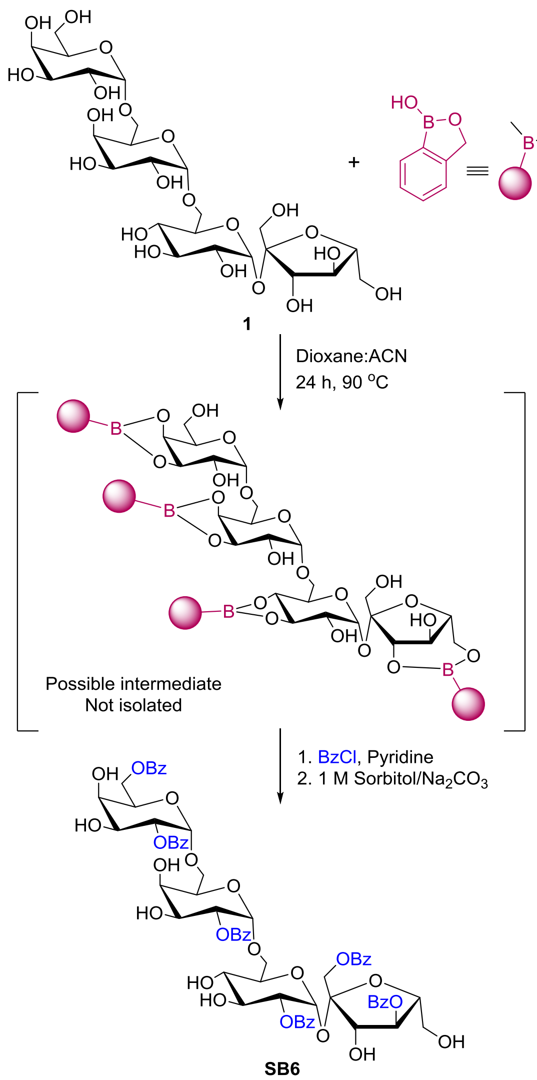

Figure S1. Indirect approach to assess the degree of complexation.

4. Mass spectrometry analysis

MALDI sample concentration: 1 mg/ml in MeOH + 5% ammonia. Matrix preparation: DHB (2,5-Dihydroxybenzoic acid); 10 mg/ml dissolved in water:acetonitrile 50:50 + 0.01% formic acid. Sample and matrix were combined in a ratio matrix:sample 10:1. The solution (1 µl) was then loaded on a MALDI plate and allowed to dry. An Nd:YAG laser (355 nm) was used with a repetition rate of 2500 Hz and a mean laser energy per pulse of 4 µJ (software setting of 250 arbitrary units). The scan time was set at 0.1 s, and the pixel size was set at 45 µm in both the x and y axes. Multiple spectra from different location across the target surface of the MALDI plate were averaged.

Depending on the number of benzoyl groups on the sugar core, it is possible to determine the number of benzoboroxole units that were bound to stachyose in the complex (Figure S1). Consequently, according to the available potential binding sites of stachyose **1**, a complex with a 1:4 stoichiometry should afford a product with six benzoyl groups **SB6**, whose possible structure is proposed in Figure S1. If less benzoboroxoles were bound, we should observe a higher number of benzoyl groups (Figure S2). Similar analysis can be extended to nystose (Figure S3) and verbascose (Figure S4).

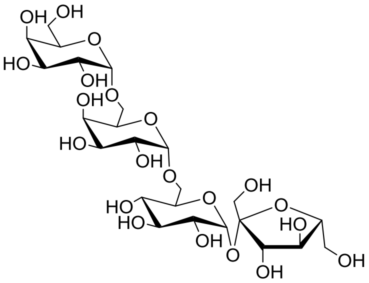

1

MW = 666.58 gmol^{-1}

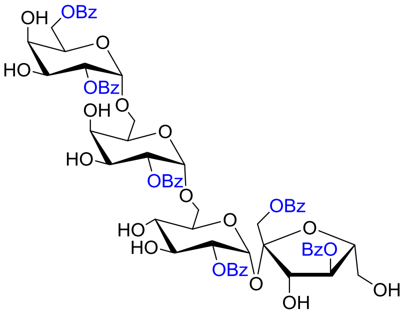

SB6

MW = 1291.23 gmol^{-1} From a 1:4 complex

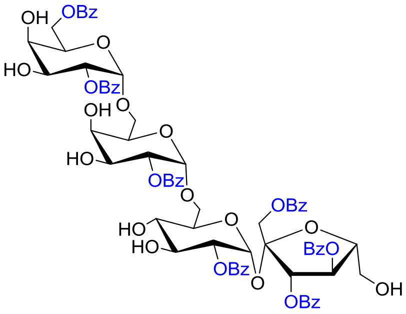

SB7

MW = 1395.34 gmol^{-1} From a 1:4 complex

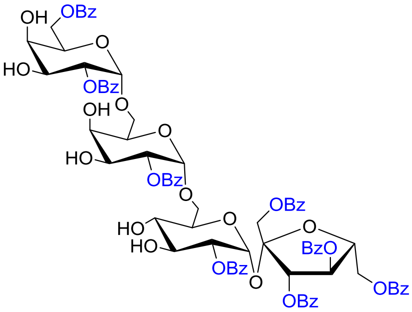

SB8

MW = 1499.44 gmol^{-1} From a 1:3 complex

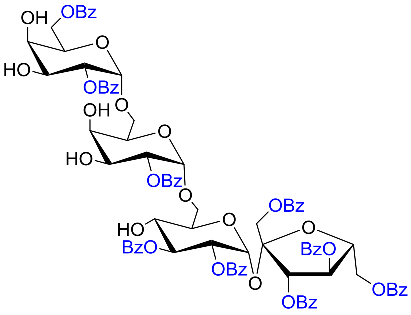

SB9

MW = 1603.55 gmol^{-1} From a 1:3 complex

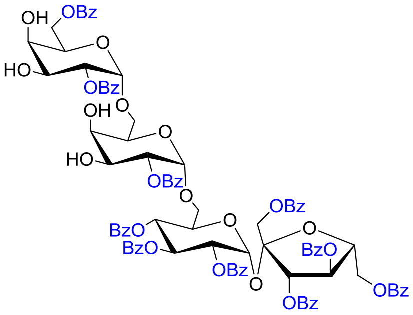

SB10

MW = 1707.66 gmol^{-1} From a 1:2 complex

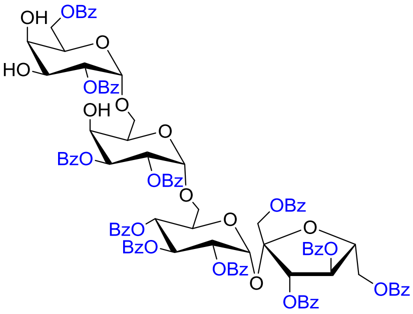

SB11

MW = 1811.77 gmol^{-1} From a 1:2 complex

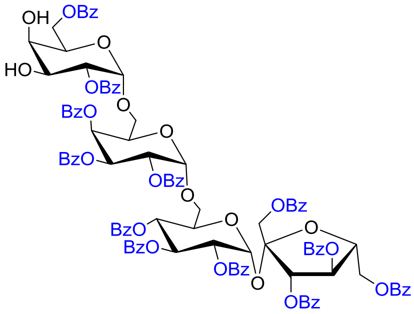

SB12

MW = 1915.88 gmol^{-1} From a 1:1 complex

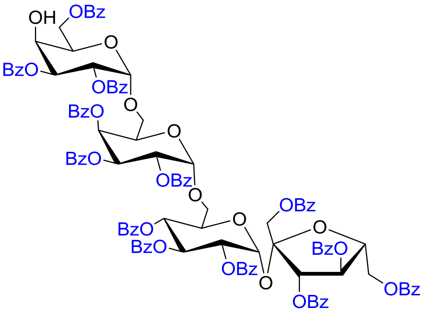

SB13

MW = 2019.98 gmol^{-1} From a 1:1 complex

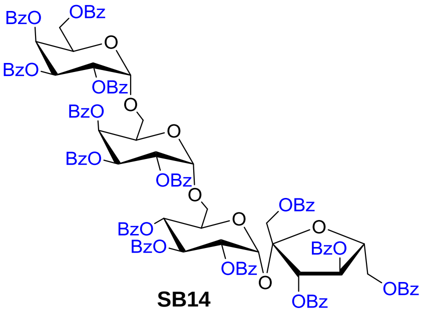

SB14

MW = 2124.09 No complex

Figure S2. Benzoylated derivatives with stachyose **1**: compounds with an odd number of benzoyl groups B derive from complexes where the boron is bound to the sugar S via only one OH group.

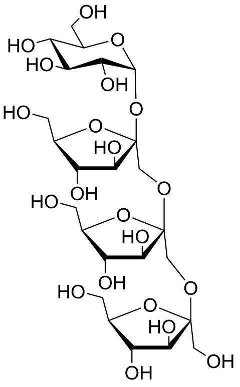

2

MW = 666.58 gmol^{-1}

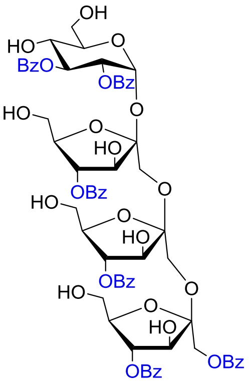

SB6

MW = 1291.23 gmol^{-1}

From a 1:4 complex

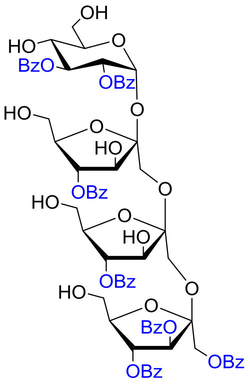

SB7

MW = 1395.34 gmol^{-1}

From a 1:4 complex

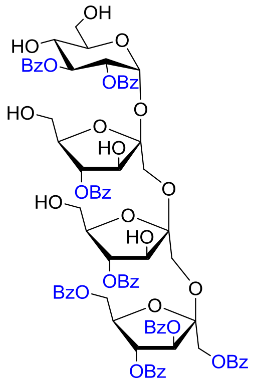

SB8

MW = 1499.44 gmol^{-1}

From a 1:3 complex

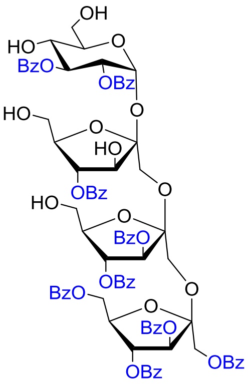

SB9

MW = 1603.55 gmol^{-1}

From a 1:3 complex

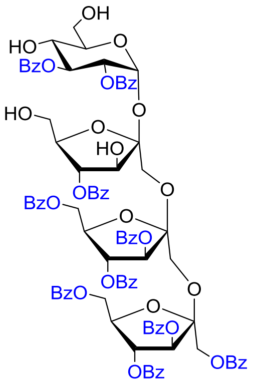

SB10

MW = 1707.66 gmol^{-1}

From a 1:2 complex

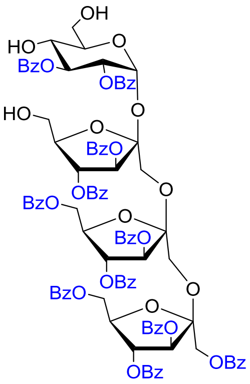

SB11

MW = 1811.77 gmol^{-1}

From a 1:2 complex

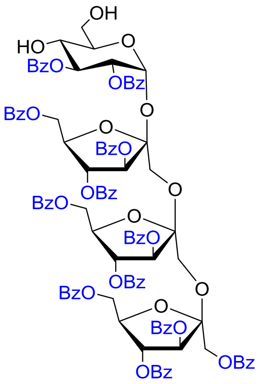

SB12

MW = 1915.88 gmol^{-1}

From a 1:1 complex

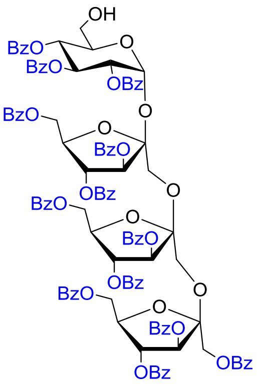

SB13

MW = 2019.98

From a 1:1 complex

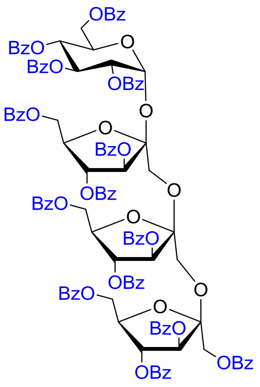

SB14

MW = 2124.09

No complex

Figure S3. Benzoylated derivatives with nystose **2**: compounds with an odd number of benzoyl groups B derive from complexes where the boron is bound to the sugar S via only one OH group.

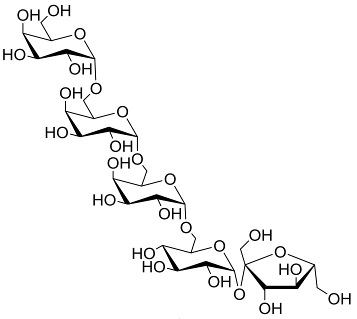

3

MW = 828.78 gmol^{-1}

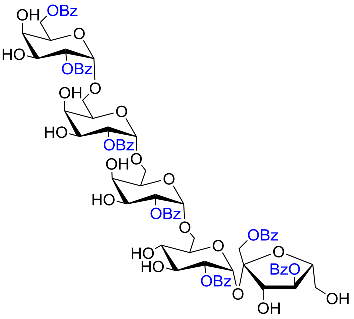

SB7

MW = 1557.48 gmol^{-1} From a 1:5 complex

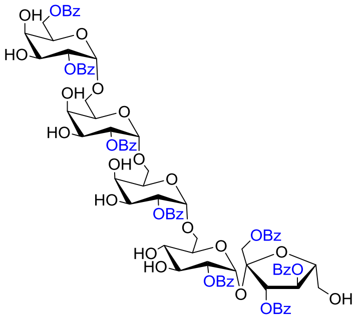

SB8

MW = 1661.58 gmol^{-1} From a 1:5 complex

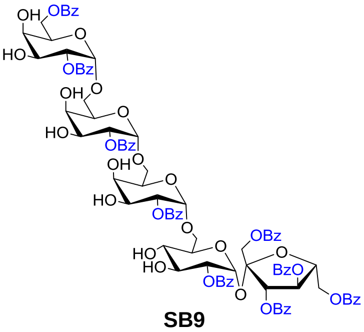

SB9

MW = 1765.69 gmol^{-1} From a 1:4 complex

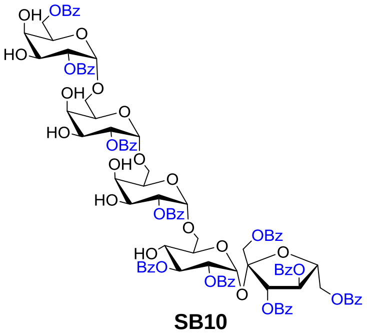

SB10

MW = 1869.80 gmol^{-1} From a 1:4 complex

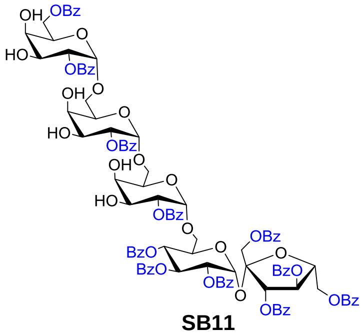

SB11

MW = 1973.91 gmol^{-1} From a 1:3 complex

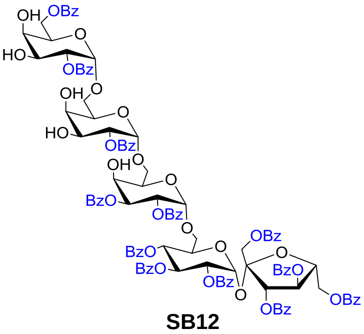

SB12

MW = 2078.02 gmol^{-1} From a 1:3 complex

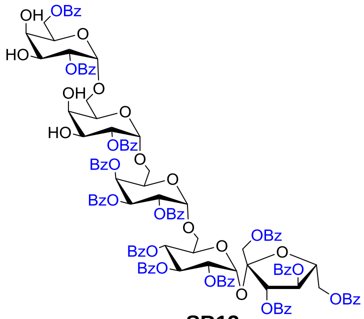

SB13

MW = 2182.12 gmol^{-1} From a 1:2 complex

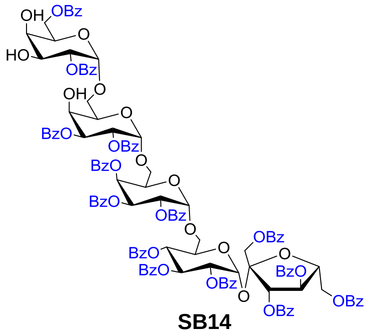

SB14

MW = 2286.23 gmol^{-1} From a 1:2 complex

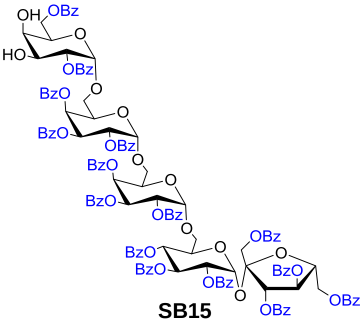

SB15

MW = 2390.34 gmol^{-1} From a 1:1 complex

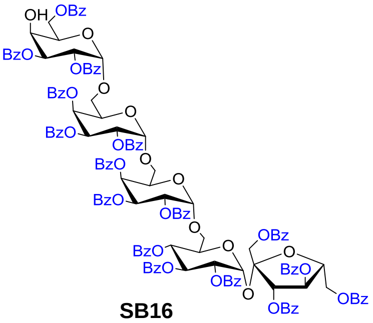

SB16

MW = 2494.45 gmol^{-1} From a 1:1 complex

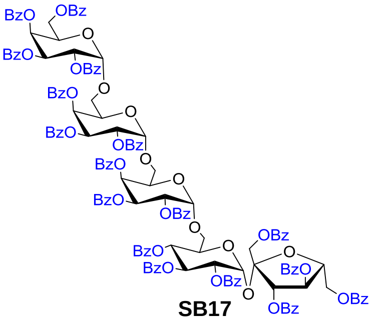

SB17

MW = 2598.56 gmol^{-1} No complex

Figure S4. Benzoylated derivatives with verbascose **3**: compounds with an even number of benzoyl groups B derive from complexes where the boron is bound to the sugar S via only one OH group.

Chromatography led to the isolation of fractions containing a mixture of two or more species, since it was not possible to isolate pure products (Figures S5-S7). In order to determine the amount of the different products in each fraction, we used the relative ratios of the peaks in the mass spectra. Although peak heights of different analytes can vary significantly, we can assume that the ionization of the benzoylated species that differ for one or two benzoyl groups is very similar. As previously described,[3] all the spectra were normalized by dividing the intensities of the individual signals by the overall sum of all the selected peaks (**SB6** to **SB9** for stachyose). In addition, to minimise variability, multiple spectra from different location across the target surface of the MALDI plate were averaged. Relative signal ratios remained quite stable. This afforded the relative amount of each species within each sample and allowed us to determine the distribution of the benzoylated oligosaccharides (Figure S8). This approach cannot be considered an entire quantitative analysis, however, it can provide useful insights about the complexation process. The results showed that, among the fractions collected, most of the benzoylated products derived from a complex where four benzoboroxoles were bound to stachyose (**SB6** and **SB7**), with those derived from a 1:3 complex (**SB8** and **SB9**) being minor products. In a similar manner, high-order complexes were formed with nystose and verbascose.

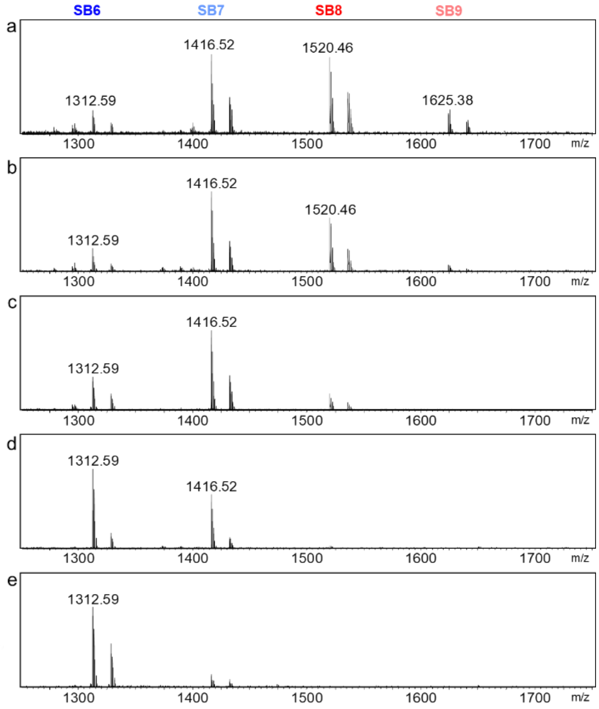

Figure S5. MALDI spectra of the fraction isolated after the complexation of stachyose **1** with 2-(hydroxymethyl)phenylboronic acid cyclic monoester followed by benzoylation (indirect approach): a) to e) fractions 1 to 5.

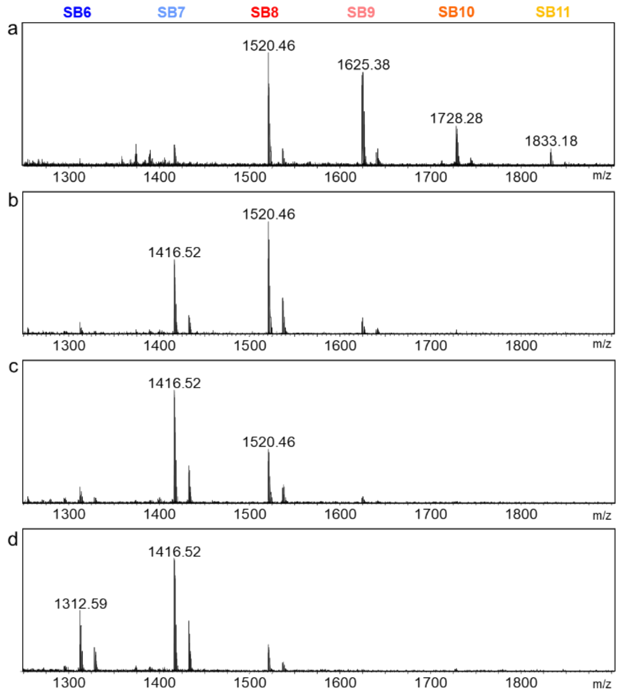

Figure S6. MALDI spectra of the fraction isolated after the complexation of nystose **2** with 2-(hydroxymethyl)phenylboronic acid cyclic monoester followed by benzoylation (indirect approach): a) to d) fractions 1 to 4.

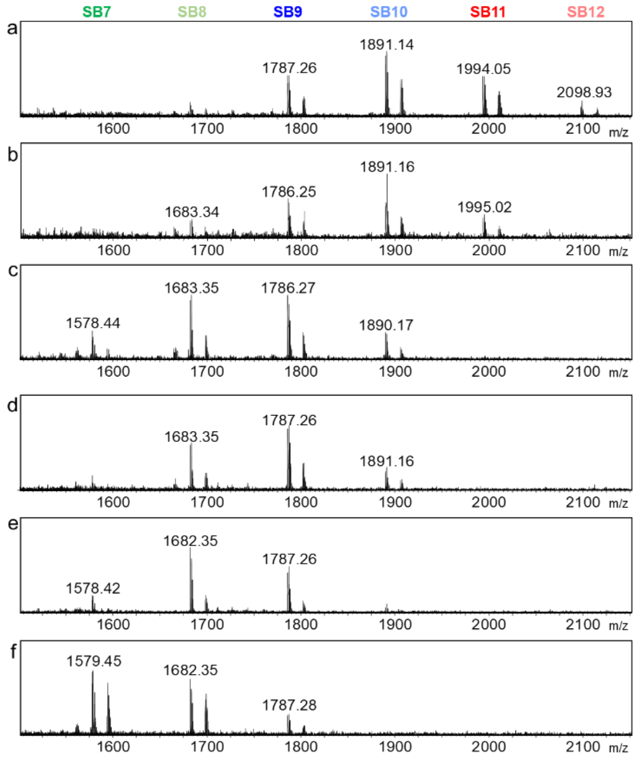

Figure S7. MALDI spectra of the fraction isolated after the complexation of verbascose **3** with 2-(hydroxymethyl)phenylboronic acid cyclic monoester followed by benzoylation (indirect approach): a) to f) fractions 1 to 6.

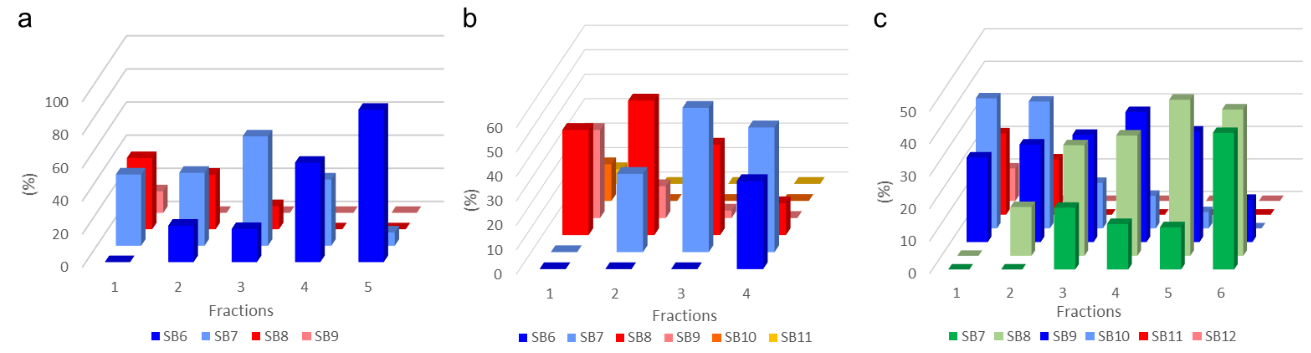

Figure S8. Distribution of the benzoylated species determined by MS analysis of the fractions isolated after the complexation of a) stachyose **1**, b) nystose **2**, c) verbascose **3** with 2-(hydroxymethyl)phenylboronic acid cyclic monoester **4** followed by benzoylation (indirect approach).

5. Self-assembled monolayer (SAM) formation and characterization

5.1 SAM Preparation

The gold substrates were cleaned by immersion in piranha solution (70% H_2SO_4, 30% H_2O_2) at room temperature for 7 minutes, rinsed with Ultra High Quality (UHQ) water and then HPLC grade ethanol thoroughly for 1 min. (*Caution: Piranha solution reacts violently with all organic compounds and should be handled with care*). Immediately after rinsing, the substrates were immersed for 24 h in ethanolic 0.1 mM solutions of *N,N'*-bis(acryloyl)cystamine containing 2% trifluoroacetic acid (TFA). The substrates were rinsed with HPLC EtOH and dried under a stream of Ar. The control benzyl-terminated SAMs were prepared in a similar way, by immersing the clean gold substrates in 0.1 mM ethanolic solutions of **5** for 24 h whilst the benzoboroxole control SAM was incubated in 0.1 mM methanolic solutions of **6**, followed by rinsing and then drying in a stream of Ar.

5.2 Contact Angle

Contact angle measurements were conducted on an Attension Theta contact angle meter from Biolin Scientific. The dynamic contact angles were recorded as an automated micro-syringe was used to add liquid onto the functionalized gold surface (advancing) or remove liquid from the drop deposited on the gold surface (receding). A video camera recorded images at an acquisition rate of 32 frames per second that were subsequently analysed using the OneAttension software to obtain the contact angles of the drops at the three-phase intersection. Averages and standard deviations were determined from six measurements for each type of SAM.

5.3 Ellipsometry

The thickness of the deposited monolayers was determined by spectroscopic ellipsometry using an Alpha-SE ellipsometer from J.A. Woollam. The ellipsometric data acquired at angles of incidence of 65°, 70° and 75° was processed using the CompleteEASE software. The thickness calculations were based on a three-phase ambient/SAM/Au model, in which the SAM was assumed to be isotropic and assigned a refractive index of 1.50. The thickness reported is the average of six measurements taken on each SAM, with the errors reported as standard deviation.

5.4 X-ray photoelectron spectroscopy (XPS)

XPS experiments were carried out using a Thermo Scientific K-Alpha XPS system and a monochromatic Al Kα X-ray source (1486.7 eV) at a take-off angle of 90° to the surface plane. High-resolution scans of C (1s), O (1s), N (1s) and S (2p) were recorded using a pass energy of 40 eV at a step size of 0.1 eV. Fitting of XPS peaks was performed using CasaXPS processing software. Sensitivity factors used in this study were: C (1s) 1.00; O (1s) 2.93; N (1s), 1.80; S (2p), 1.68. The S doublet was constrained to have a peak separation of 1.18 eV, a 2:1 area ratio ($2p_{3/2}$: $2p_{1/2}$) and equal FWHM.

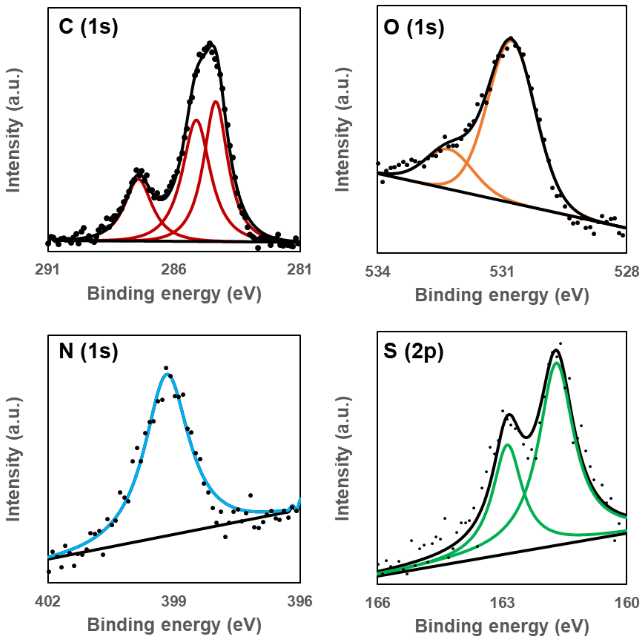

Figure S9. XPS spectra of the C (1s), O (1s), N (1s) and S (2p) regions for *N',N'*-bis(acryloyl)cystamine SAM.

6. Formation of binding scaffold and surface plasmon resonance studies

6.1 Complex formation

In a 25 ml round bottom glass flask, stachyose or nystose (6.67 mg, 10.01 µmol) and 5-acrylamido-2-(hydroxymethyl)phenylboronic acid cyclic monoester (65.0 mg, 320 µmol, 8.0 eq/sugar unit) were suspended in a mixture of dioxane (2 ml) and acetonitrile (0.34 ml). Man5 (8.29 mg, 10.01 µmo l) and 5-acrylamido-2-(hydroxymethyl)phenylboronic acid cyclic monoester (81.2 mg, 400 µmol, 8.0 eq/sugar unit) were suspended in a mixture of dioxane (2.49 ml) and acetonitrile (0.41 ml). The reaction mixture was stirred at 90 °C for 24 h under argon atmosphere in presence of activated molecular sieves 3Å. The solvent was removed under reduced pressure and the crude was treated with acetonitrile (0.6 ml) in order to afford a white precipitate. The suspension was transferred in a 1 ml Eppendorf and centrifuged. The supernatant was collected and the precipitate was re-suspended in acetonitrile (0.6 ml) for a second centrifugation. Subsequently, the supernatant was removed, the precipitate was dried and dissolved in a 1.6 ml solution of phosphate buffer (pH 7.4).

6.2 Formation of binding scaffold

The gold substrates functionalized with *N',N'*-bis(acryloyl)cystamine SAMs were added to individual polymerisation solutions containing 800 µL of the complex prepared as described in section 6.1, 100 µL of ammonium persulfate (40 mg/mL) and 100 µL of a 5 M *N,N'*-methylenebis(acrylamide) solution. 1 µL of tetramethylethylenediamine (TEMED) was subsequently added to initiate the polymerization under argon. After 15 min, the modified gold surfaces were removed from the solution, rinsed with UHQ water, ammonium acetate (pH 6), ethanol and dried under a stream of argon. Bisacrylamide only studies were conducted with 800 µL of a 0.1 M phosphate buffer saline solution, 100 µL ammonium per sulphate (40 mg/mL) and 100 µL of a 5 M *N,N'*-methylenebis(acrylamide) before adding 1 µL TEMED for 15 minutes.

6.3 Surface Plasmon Resonance (SPR)

The SPR experiments were performed on a Reichert SR7000DC Dual Channel Spectrometer (NY, USA) at 25°C. A baseline was established for each surface by running degassed 0.1 M ammonium acetate at pH 10 over the sample at 25 µl/min until baseline stabilization was achieved. SPR sensorgrams were acquired by injecting solutions of either an oligosaccharide or protein diluted in the running buffer (i.e. 1 M ammonium acetate at pH 10) for 2 min at a flow rate of 25 µl/min, following by 5 min dissociation in the running buffer and 5 min regeneration using 1 M ammonium acetate at pH 6. For each concentration, 2 measurements from 2 individual chips were taken (n=4) from which the average and standard deviation values were then calculated.
Data sets were analysed using Scrubber 2 (BioLogic Software, Campbell, Australia). In order to correct for bulk refractive index contributions arising from the oligosaccharides and proteins, SPR responses from the control 3,3'-disulfanediylbis(*N*-phenylpropanamide) (**5**) SAM were subtracted from those obtained from the surfaces containing the oligosaccharide-binding sites. The corrected SPR responses at equilibrium (Req) were plotted against the concentration of the injected oligosaccharide or protein (C_p) and fitted to a 1:1 steady-state model using Scrubber 2. The model uses a non-linear least-squares regression method to fit data to the Langmuir adsorption isotherm (Equation S1), with K_D being the dissociation constant and R_{max} the maximum analyte binding capacity of the surface.

$$R_{eq} = \left(\frac{C_p}{C_p + K_D}\right) R_{max} \qquad \text{Equation S1}$$

References

[1] M. Lin, G. Chen, M. Jiang, Polym. Chem. 2014, 5, 234.
[2] S. N. Lewis, G. A. Miller, M. Hausman, E. C. Szamborski, Journal of Heterocyclic Chemistry 1971, 8, 571.
[3] A. Schinkovitz, S. Boisard, I. Freuze, J. Osuga, N. Mehlmer, T. Bruck, P. Richomme, Anal Bioanal Chem 2018, 6187.
